# Angiopoietin-Like 3 Induces Podocyte F-Actin Rearrangement through Integrin **α**_*V*_ **β**_3_/FAK/PI3K Pathway-Mediated Rac1 Activation

**DOI:** 10.1155/2013/135608

**Published:** 2013-11-05

**Authors:** Yi Lin, Jia Rao, Xi-liang Zha, Hong Xu

**Affiliations:** ^1^Department of Nephrology and Rheumatism, Children's Hospital of Fudan University, Shanghai 201102, China; ^2^Department of Pediatrics, Affiliated Hospital of Medical College, Qingdao University, Shandong 266003, China; ^3^Department of Biochemistry and Molecular Biology, Shanghai Medical College, Fudan University, Shanghai 200032, China

## Abstract

Glomerular podocytes are highly differentiated cells whose foot processes, which are mainly maintained by the architecture of actin filaments, have a unique morphology. A rearrangement of F-actin in podocytes causes changes in their motility that involve foot process effacement and proteinuria in glomerular diseases. Members of the Rho family small GTPases, especially RhoA, Rac1, and Cdc42, are key molecules in the regulation of actin cytoskeleton rearrangement. Our previous study showed that angiopoietin-like 3 (Angptl3) can increase the motility of podocytes *in vitro*. In this study, we found that recombinant Angptl3 treatment, together with the activation of Rac1, could cause F-actin rearrangement in podocytes. We also found that these effects could be blocked by an integrin *α*_*V*_ *β*_3_ inhibitor, implicating integrin *α*_*V*_ *β*_3_ as the Angptl3 receptor in its effects on actin cytoskeleton rearrangement. In addition, we studied the molecular pathway for this process. Our results showed that in podocytes, Angptl3 could induce actin filament rearrangement, mainly in lamellipodia formation, and that this process was mediated by integrin *α*_*V*_ *β*_3_-mediated FAK and PI3K phosphorylation and Rac1 activation. Our results might provide a new explanation for the effect of Angptl3 on increasing podocyte motility.

## 1. Introduction

Significant proteinuria is a characteristic manifestation of glomerular filtration barrier damage; moreover, long-term significant proteinuria itself is an important risk factor that can lead to chronic kidney disease and even to end-stage renal disease [1]. The ultrastructure of glomeruli in nearly all glomerular proteinuria diseases shows diffused foot process effacement, indicating the important role of podocytes in the generation of proteinuria. 

Podocytes in the glomerulus form the outer layer of the glomerular filtration barrier and play an important role in maintaining its permselectivity [2]. Podocytes are unique cells with a complex cellular organization that is characterized by foot processes. Adjacent foot processes form an interdigitating pattern, and the space in between the filtration slits is known as slit diaphragm, which is bridged by podocyte membrane molecules.

The special morphology of podocytes is based on an underlying network of dynamic and interconnected actin and microtubule polymers. Podocyte foot processes have a subcortical network of branched actin filaments as well as bundled filaments that run longitudinally through the process [3]. In glomerular diseases, a rearrangement of F-actin leads to the alterations in podocyte motility, which are considered to underlie foot process effacement and proteinuria [4]. 

Actin filaments can exist as networks or bundles that are highly regulated by more than 100 proteins. In podocytes, this dynamic regulation is mainly modulated by membrane proteins that are located in the glomerular basement membrane and slit diaphragm areas [5]. Integrins are the primary molecules related to actin filament regulation in podocyte basement membrane area [6]. Rho family small GTPases are important proteins that regulate cytoskeletal dynamics, among which RhoA, Rac1, and Cdc42 are the most important, and serve as molecular switches that relay signals from the membrane to the cytoskeleton [7].

Angiopoietin-like 3 (Angptl3) is a secreted protein that is mainly expressed in hepatocytes and is weakly expressed in normal kidney cells [8]. Studies have shown that Angptl3 secreted by hepatocytes plays an important role in the regulation of lipid metabolism as a powerful inhibitor of lipoprotein lipase [9]. However, little is known about its physiological and pathological function in the kidney. Additionally, podocyte-secreted Angptl4, which is highly homologous to Angptl3 [10], has been implicated in proteinuria in glucocorticoid-sensitive nephrotic syndrome [11]. Until now, few studies have focused on the relationship between Angptl3 and proteinuria.

In our previous study, we found that Angptl3 expression was upregulated in nephrotic syndrome kidney tissue [12]. In addition, Angptl3 was concentrated in glomerular podocytes in both humans and rats [13]. Another study revealed that the altered expression of Angptl3 in the glomerulus was associated with proteinuria and foot process effacement in kidney diseases [14]. Moreover, we found that up-regulating podocyte Angptl3 expression *in vitro* increases podocyte motility [15]. 

To explore Angptl3's function in podocytes, we investigated the effect of Angptl3 on the rearrangement of podocyte F-actin and the activation of small GTPases. In this study, we found that Angptl3 could lead to podocyte F-actin rearrangement and Rac1 activation, which is induced through integrin *α*_*V*_ *β*_3_ and is mediated by FAK and PI3K phosphorylation.

## 2. Materials and Methods

### 2.1. Reagents and Antibodies

Recombinant mouse Angptl3 was obtained from R&D systems (Minneapolis, MN, USA). Rac1 inhibitor NSC23766 was purchased from the Cayman Chemical Company (Ann Arbor, MI, USA). RhoA inhibitor CT04 was purchased from Cytoskeleton (Denver, CO, USA). Integrin *α*_*V*_ *β*_3_ inhibitor echistatin *α*1 isoform was purchased from Tocris (Bristol, UK). FAK Inhibitor 14 was purchased from Santa Cruz Biotechnology (Santa Cruz, CA, USA). PI3K inhibitor LY294002 was purchased from Beyotime Biotechnology Corporation (Shanghai, China). Fluorescein isothiocyanate- (FITC-) labeled phalloidin was from Sigma-Aldrich (St. Louis, MO, USA). G-LISA RhoA, Rac1, and Cdc42 activation assay kits were purchased from Cytoskeleton Technology. Monoclonal antibodies to RhoA, Rac1, Cdc42, FAK, and PI3K were purchased from Cell Signaling Technology (Beverly, MA, USA). The monoclonal antibody to phosphorylated FAK was purchased from BD (San Diego, CA, USA). The polyclonal antibody to phosphorylated PI3K was purchased from Signalway Antibody (Pearland, TX, USA). The monoclonal antibody to glyceraldehyde phosphate dehydrogenase (GAPDH) was purchased from KangChen Bio-tech (Shanghai, China). Horseradish peroxidase- (HRP-) conjugated secondary antibodies were purchased from Cell Signaling Technology.

### 2.2. Cell Lines, Culture, and Treatment

The mouse podocytes used in this study were a conditionally immortalized cell line originated by Dr. Peter Mundel (Massachusetts General Hospital, Boston, MA, USA). Cells were cultured and differentiated as previously described [16]. Briefly, podocytes were cultured in RPMI 1640 medium (Thermo, Beijing, China) containing 10% fetal bovine serum (FBS, Gibco, Grand Island, NY, USA), 100 U/mL penicillin, and 100 *μ*g/mL streptomycin. Podocytes were cultured by growth in medium containing 10 U/mL mouse INF-*γ* (ProSpec, Israel) at 33°C with 100% relative humidity and a 5% CO_2_ atmosphere, and they were induced to differentiate by culture at 37°C in medium without INF-*γ* for 10 to 14 days. Podocytes became large cells with plenty of small branches when well differentiated. All cell culture dishes were coated with type I collagen (Sigma-Aldrich), and cells never reached 90% confluency.

The cells were starved with 0.5% FBS culture media for 1 day when they reached 50–60% confluency and were then serum-depleted overnight, treated with or without inhibitors, and finally treated with 250 ng/mL Angptl3 for an appropriate time before immunofluorescent staining, western blot, or G-LISA assay.

### 2.3. G-LISA Assay

RhoA, Rac1, and Cdc42 activation in podocytes was determined with the G-LISA RhoA, Rac1 and Cdc42 activation assays, respectively. The G-LISA assay is a method that measures the quality of GTP-bound, or activated, small GTPases in cell or tissue samples. After treatment, cells were washed with PBS, resuspended in kit lysis buffer and harvested with a cell scraper. The total protein concentration in each lysate was determined by the Precision Red advanced protein assay reagent in the kits. The G-LISA kits contain a RhoA, Rac1, or Cdc42 GTP-binding protein that is immobilized on the provided microplates. Bound active small GTPases were treated with a specific primary antibody and then with horseradish peroxidase- (HRP-) conjugated secondary antibody and HRP detection reagent. The optical density was measured in a Tecan M200 plate reader (Tecan, Research Triangle Park, NC, USA) at 490 nm.

### 2.4. Immunofluorescence Staining

Cells were seeded on 12 mm glass coverslips precoated with type I collagen in a 24-well cluster plate and treated as described above. Cells were then washed twice with PBS and fixed in 4% paraformaldehyde in PBS. The cells were then incubated in 4°C overnight with FITC-labeled phalloidin to stain F-actin and for 5 min with DAPI to stain the nucleus. Coverslips were mounted with antiquench mounting medium. Images were captured on an Olympus IX81 fluorescence microscope (Olympus, Japan) at 400x magnification.

### 2.5. Lamellipodia Quantification

Cellular lamellipodia which were defined as the projections that arise from the cell body, including well-defined typical membrane ruffles and finger-like protrusions in the shape of smooth bumps or spikes, were counted. Filopodia were not included in this count. In each group, 10 visual fields were randomly selected, and altogether, 100 cells were analyzed. The results are expressed as the number of lamellipodia per cell.

### 2.6. Western Blots

The cells were washed with TBS and lysed in a buffer containing 50 mM Tris-HCl (pH 6.8), 2% SDS, 10% glycerol, phosphatase inhibitors (100 mM Na_3_VO_4_ and 10 mM NaF), and protease inhibitors (1 mM PMSF), and then the lysates were quantified using the Enhanced BCA Protein Assay Kit (BioTime, Shanghai, China). Equal amounts of lysates (30 *μ*g) were loaded on a 10% SDS-PAGE gel and blotted onto PVDF membranes (Millipore Corp.). The samples were blocked in TBS-Tween (TBS-T) (20 mM Tris-HCl, pH 7.4; 0.05% Tween 20) with 5% nonfat dry milk, and the membranes were incubated with primary antibodies at appropriate dilutions in 5% milk TBS-T overnight at 4°C. Subsequently, the membranes were washed three times with TBS-T solution, followed by an incubation with HRP-conjugated secondary antibody (1 : 3000) in TBS-T and 5% milk. The ECL signals were captured using a CCD camera (ImageQuant LAS 4000 mini, GE Healthcare, Sweden).

### 2.7. Statistical Analysis

All experiments were repeated at least three times. The values, expressed as means ± SEM, were subjected to Student's *t*-test or one-way ANOVA followed by a Student Newman-Keuls post hoc test, with *P* values of <0.05 considered statistically significant.

## 3. Results and Discussion

For many years, studies on proteinuria concentrated on the function of podocyte structural proteins and the genes that encode them, the mutations of which are the main cause of inherited nephrotic syndrome [2]. But most nephrotic syndrome patients, especially children, who do not have such gene defects also show significant proteinuria and foot process effacement, indicating that there are other factors involved in the generation of proteinuria. In recent studies, many soluble factors, especially those secreted by podocytes, were discovered and found to interfere with podocyte function and lead to proteinuria. This leads to new studies on the development of proteinuria [11, 17].

 The Angptl3 expression of podocytes is greatly increased in proteinuria diseases, showing that Angptl3 may be involved in the generation of proteinuria. We have found that Angptl3 expression can increase podocytes motility [15], which suggests that Angptl3 may induce F-actin rearrangement in podocytes. In this study, we tested a signaling pathway for this process.

### 3.1. Angptl3 Stimulated the F-Actin Rearrangement That Causes a Particular Lamellipodia Formation in Podocytes

**Figure 1:**
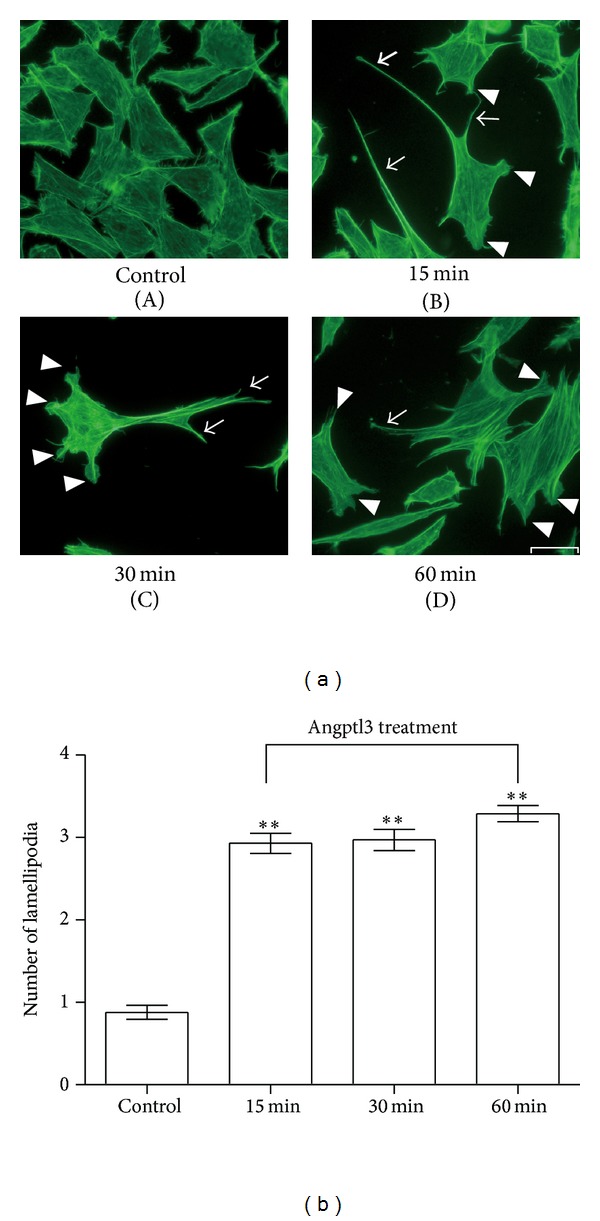
Angptl3-induced actin filament rearrangement in podocytes, characterized by lamellipodia formation. (a) F-actin in podocytes after treatment with Angptl3 (250 ng/mL) for 15 min, 30 min, and 60 min versus control. Scale bar, 20 *μ*m. Arrows in the pictures show the lamellipodia formation. Arrowheads show cell spikes. Pictures are representative of all the results obtained in the different experimental conditions. (b) Comparison of podocyte lamellipodia formation between groups (*n* = 100, control group versus Angptl3 treatment groups, **P < 0.01).

Actin filaments are important for maintaining normal podocyte morphology and motility [4]. To investigate whether Angptl3 plays an important role in F-actin rearrangement in podocytes, we treated podocytes with recombinant Angptl3 for 15 min, 30 min, and 60 min, stained F-actin with FITC-labeled phalloidin, and observed the cells through fluorescence microscopy. We found that after Angptl3 treatment, the podocytes formed more spikes and lamellipodia (Figure 1(a)) compared with the control group. The comparison of the number of lamellipodia between the Angptl3 treatment groups and the control group was statistically significant, while there was no difference between the Angptl3 treatment groups (Figure 1(b)). It has been widely accepted that the lamellipodia formation is related to an increase in cell motility [18], which is consistent with our previous result [15]. 

### 3.2. The Lamellipodia Formation Induced by Angptl3 Was Rac1 Dependent in Podocytes

Rho family small GTPases, especially RhoA, Rac1, and Cdc42, are key molecules in the control of F-actin reorganization. The Rho switch operates by alternating between an active, GTP-bound state and an inactive, GDP-bound state. These three small GTPases play different roles in regulating the F-actin cytoskeleton; in brief, the activation of RhoA leads to stress fiber formation that is believed to stabilize the actin cytoskeleton, while the activation of Rac1 and Cdc42 leads to lamellipodia and filopodia formation, respectively; these are important for increasing cell motility. In podocytes, small GTPases are also believed to play a key role in cytoskeleton regulation and are involved in the formation and development of proteinuric diseases [4]. Therefore, we tested whether small GTPases can be activated by Angptl3 in podocytes. 

**Figure 2:**
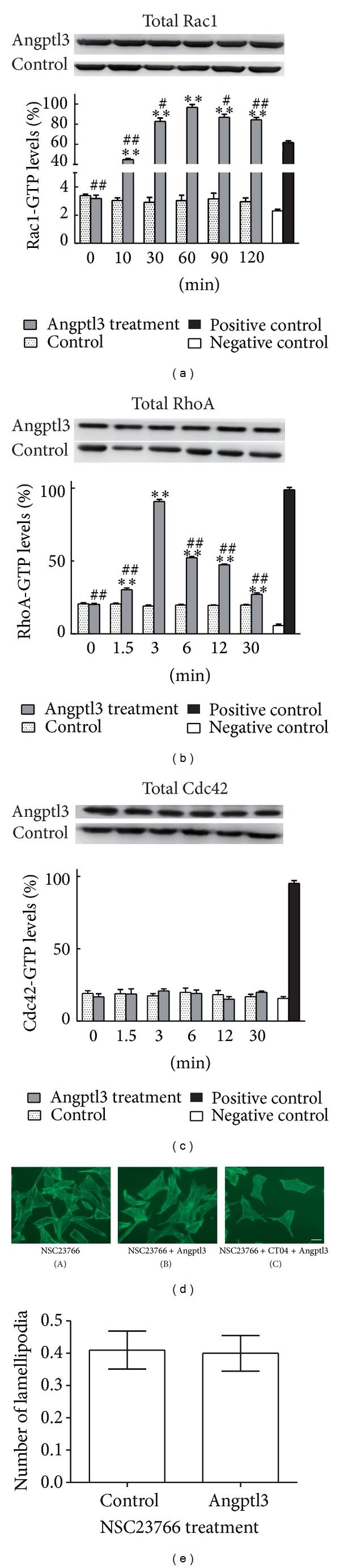
Activation of Rac1 and RhoA, especially Rac1, played a central role in podocyte actin filament rearrangement induced by Angptl3. (a), (b), and (c) Changes in the activation of small GTPases Rac1, RhoA, and Cdc42 after treatment with Angptl3 (250 ng/mL) for different times. The relative total small GTPase protein levels were detected by western blot and are shown in the top panel. The levels of the active forms of the small GTPases were detected with G-LISA and are shown in the bottom panel. No lysate was added in negative control groups, while control protein instead of lysate was added in positive control groups. Compared to the control group, *P < 0.05, **P < 0.01. Compared to the peak value, ^#^ *P* < 0.05, ^##^ *P* < 0.01. (d) Lamellipodia formation in podocytes after blocking Rac1, followed by Angptl3 treatment. (A) F-actin in podocytes incubated with 80 mM NSC23766 for 2 h. (B) F-actin in podocytes preincubated with 80 mM NSC23766 for 2 h, followed by 250 ng/mL Angptl3 treatment for 30 min. (C) F-actin in podocytes preincubated with 80 mM NSC23766 and 80 mM CT04 for 2 h, followed by 250 ng/mL Angptl3 treatment for 30 min. Scale bar, 20 *μ*m. The comparison of lamellipodia formation between groups (A) and (B) is shown in (e).

The G-LISA analyses (Figures 2(a) and 2(b)) showed that both Rac1 and RhoA were activated after Angptl3 treatment, but the activation of Rac1 and RhoA reached peaks at different times. Additionally, the activation of Rac1 lasted up to 120 min, and this is much longer than that of RhoA, which returned to its basal level in 30 min. The activation of RhoA may help in maintaining normal stress fibers in podocytes, but this phenomenon was weak and transient. By contrast, the activation of Rac1 was much stronger, suggesting Rac1 as the main Angptl3-induced factor interfering with F-actin rearrangement. Cdc42 was not activated upon treatment with Angptl3 (Figure 2(c)). These results indicate that after Angptl3 treatment in podocytes, lamellipodia should increase, which matches well with the previously observed phenomenon.

To investigate whether Rac1 activation is the main cause of Angptl3-mediated lamellipodia formation, we pre-incubated podocytes with Rac1 inhibitor NSC23766, which can block the effect of Rac1 without interfering with the effects of RhoA and Cdc42 [19], for 2 h and then treated cells with Angptl3. The lamellipodia formation was inhibited and stress fiber formation increased (Figures 2(d) and 2(e)).

According to the G-LISA results, RhoA could also be activated by Angptl3 treatment in podocytes, which indicated that an increase in stress fiber formation may occur. But in untreated podocytes, the background of actin filaments was strong, so a slight increase in stress fiber formation might be difficult to detect. After blocking Rac1 with NSC23766, followed by Angptl3 treatment, we found both a decrease in lamellipodia formation and a strong increase in stress fiber formation. If RhoA and Rac1 were both inhibited with CT04 and NSC23766 administered together, lamellipodia formation and stress fiber formation failed to be induced following Angptl3 treatment. This indicated that podocyte stress fiber formation induced by Angptl3 was mediated through RhoA activation. A previous study from Attias et al. proved that Rac1 activation can inhibit RhoA activation in podocytes [20]; this might explain why RhoA was only activated for a short time and why stress fiber formation did not greatly increase after Angptl3 stimulation. We can see that Rac1 and RhoA, especially Rac1, are key small GTPases in the regulation of actin cytoskeleton reorganization in Angptl3-treated podocytes and in the increase in lamellipodia and cell spike formations.

### 3.3. Integrin *α*_*V*_ *β*_3_, the Cell Surface Receptor of Angptl3, Mediated F-Actin Rearrangement through Small GTPases Rac1 and RhoA

Angptl3 is composed of two domains, which are the C-terminal fibrinogen-like domain (FLD) and the N-terminal coiled-coil domain (CCD). As Camenisch et al. [21] have reported, integrin *α*_*V*_ *β*_3_ is a previously known surface receptor of Angptl3 that can bind to the FLD of Angptl3 and play a role in vessel formation in endothelial cells. Integrin *α*_3_ *β*_1_ is the major podocyte integrin that mediates podocyte's adhesion to GBM [22]. Podocytes also express integrin *α*_*V*_ *β*_3_ on their surface, and the activation of this integrin is believed to induce proteinuria through small GTPase activation and by affecting podocyte motility [17]. We explored whether Angptl3-induced F-actin rearrangement is mediated by integrin *α*_*V*_ *β*_3_. 

**Figure 3:**
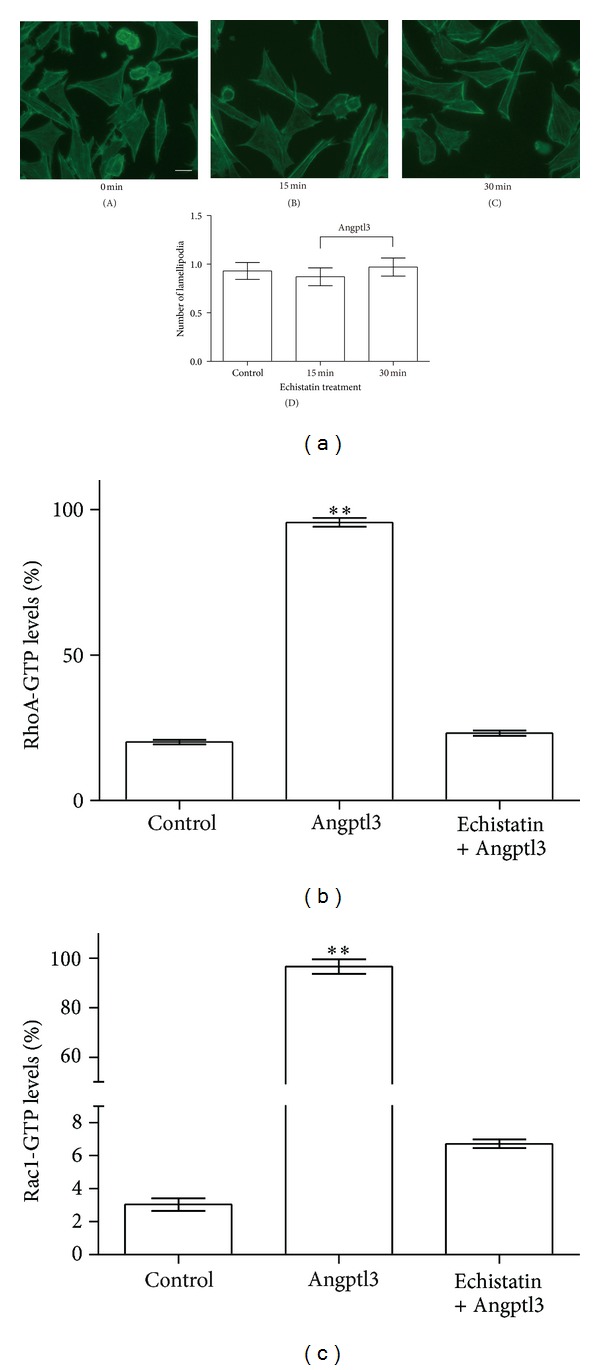
Blocking integrin *α*_*V*_ *β*_3_ blocked actin filament rearrangement and Rac1 and RhoA activation as induced by Angptl3. (a) F-actin in podocytes preincubated with 0.1 *μ*M echistatin *α*1 isoform for 1 h, followed by treatment with 250 ng/mL Angptl3 for 15 min (B), 30 min (C), or no treatment (A). Scale bar, 20 *μ*m. The comparison of lamellipodia formation between groups is shown in (D). (b) Comparison of RhoA activation between podocytes pretreated with echistatin or left untreated. Compared to control group, *P < 0.05, **P < 0.01. (c) Comparison of Rac1 activation between podocytes pretreated with echistatin or left untreated. Compared to control group, *P < 0.05, **P < 0.01.

Podocytes were treated with an integrin *α*_*V*_ *β*_3_ inhibitor, echistatin *α*1 isoform, for 1 hour then followed by treatment with Angptl3. The podocyte F-actin rearrangement disappeared (Figure 3(a)). Meanwhile, GTPase activation analyses also showed a clear inhibition of Rac1 and RhoA activation (Figures 3(b) and 3(c)). These results demonstrated that Angptl3 induced F-actin rearrangement through small GTPases Rac1 and RhoA via integrin *α*_*V*_ *β*_3_.

### 3.4. Integrin-Mediated FAK/PI3K Phosphorylation Is Important for Rac1 Activation and Lamellipodia Formation

Focal adhesion kinase (FAK) is an intracellular nonreceptor tyrosine kinase and an important modulator of integrin-dependent focal cell contacts, thereby orchestrating well-known integrin-initiated outside-in signaling events. FAK colocalizes with *β*-integrin, becomes activated by autophosphorylation at position Y-397, then performs downstream signaling events, and plays critical roles in cell motility. A previous study [23] has shown that FAK is expressed in podocytes and is activated in many podocyte pathological processes. Meanwhile, inhibiting FAK can reduce podocyte motility *in vitro* and alleviate proteinuria *in vivo*. 

**Figure 4:**
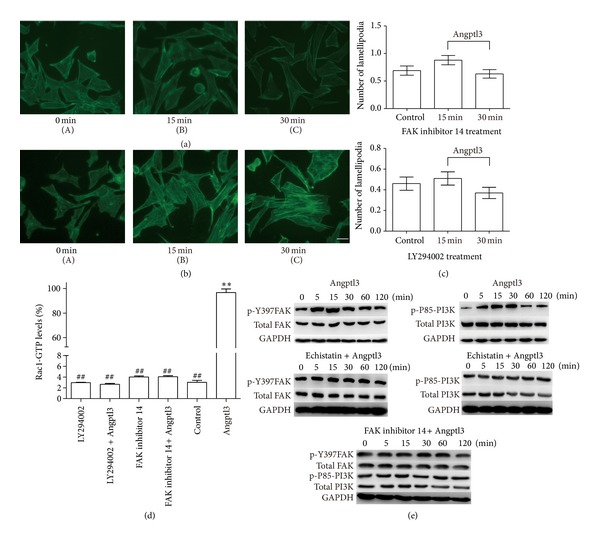
Angptl3-integrin *α*_*V*_ *β*_3_-induced podocyte lamellipodia and Rac1 activation via FAK-PI3 K phosphorylation. (a) F-actin in podocytes preincubated with 50 *μ*M FAK inhibitor 14 for 1 h, followed by 250 ng/mL Angptl3 treatment for 15 min (B), 30 min (C), or no treatment (A). (b) F-actin in podocytes pre-incubated with 50 *μ*M LY294002 for 0.5 h, followed by 250 ng/mL Angptl3 treatment for 15 min (B), 30 min (C), or no treatment (A). Scale bar, 20 *μ*m. The comparison of lamellipodia formation between groups in (a) and (b) is shown in (c). Cells were not treated in the control group, were treated with 50 *μ*M FAK inhibitor 14 or 50 *μ*M LY294002 for 1 h in FAK inhibitor 14 group and LY294002 group, were treated with 250 ng/mL Angptl3 for 1 h in the Angptl3 group, or were treated with 50 *μ*M FAK inhibitor 14 or 50 *μ*M LY294002 for 1 h, followed by 250 ng/mL Angptl3 treatment for 1 h in FAK inhibitor 14 + Angptl3 group and LY294002 + Angptl3 group. The comparison of Rac1 activation levels between groups is shown in (d). **Compared to control group, *P* < 0.01. ^##^Compared to Angptl3 group, *P* < 0.01. (e) Phosphorylation of Y397FAK and p85-FAK in podocytes pre-incubated with echistatin, LY294002, or no inhibitor, followed by Angptl3 treatment.

The next experiment was therefore to examine whether the inhibition of FAK can block the effect of Angptl3 on F-actin rearrangement in podocytes. We treated cultured podocytes with FAK inhibitor 14, which can inhibit FAK activation by preventing the phosphorylation of FAK on Y-397 [24], for 1 hour, and then treated the cells with Angptl3. The result was that a rearrangement of F-actin did not occur (Figure 4(a)). The G-LISA assay also showed a total inhibition of the activation of RhoA and Rac1 (Figure 4(c)). These results indicated that FAK activation is essential for Angptl3-mediated F-actin rearrangement and small GTPase Rac1 and RhoA activation in podocytes.

Phosphoinositide 3-kinase (PI3K) is a classic downstream target of FAK and is composed of two subunits, of which the phosphorylation of the p85 subunit is important to its activation [25]. PI3K can mediate cell motility through the activation of small GTPases. It has been reported that PI3K activation is involved in some renal disease models and that a PI3K inhibitor can alleviate kidney damage [26].

We then used the PI3K inhibitor LY294002 to block PI3K and investigate whether it interferes with the effect of Angptl3 on podocyte morphology and small GTPase activation. Our result showed that the lamellipodia formation process was stopped, but stress fiber formation was unaffected (Figures 4(b) and 4(c)). The G-LISA assay showed that after LY294002 treatment, the activation of Rac1 stimulated by Angptl3 was totally inhibited (Figure 4(d)). These results showed that PI3K activation is essential for Angptl3-mediated Rac1 activation. 

Next, we explored the possible signal pathway by which Angptl3- integrin *α*_*V*_ *β*_3_ regulated the activities of RhoA or Rac1. Since Rac1 activation and lamellipodia formation were the main effects of Angptl3 expression in podocytes, we mainly studied the signaling pathway of this process. The inhibition of FAK or PI3K blocked Angptl3-induced Rac1 activation, and both factors were involved in this process.

We stimulated podocytes with Angptl3 and found that the levels of p-Y397 FAK and p-P85 PI3K both increased (Figure 4(e)). We then inhibited the key molecules in this process by preincubating podocytes with echistatin isoform *α*1 or FAK inhibitor 14, respectively, treated cells with Angptl3, and detected the phosphorylation of possible downstream molecules. We found that after the treatment with echistatin isoform *α*1, the phosphorylation of FAK and PI3K was totally inhibited, and after treating cells with FAK inhibitor 14, PI3K phosphorylation was inhibited. These results showed that cell signals pass through Angptl3, integrin *α*_*V*_ *β*_3_, and FAK to activate RhoA, and they may pass through Angptl3, integrin *α*_*V*_ *β*_3_, FAK, and PI3K to activate Rac1 in podocytes.

To summarize, our research provided an explanation for the phenomenon that Angptl3 can increase the motility of podocytes *in vitro*. Our results indicated that Angptl3 may bind integrin *α*_*V*_ *β*_3_ on the surface of podocytes and in turn cause the phosphorylation of downstream molecules FAK and PI3K to active Rac1, which leads to the formation of spikes and lamellipodia in podocytes. Simultaneously, the phosphorylation of FAK may also activate another Rho family small GTPase, RhoA, which can lead to stress fiber formation in podocytes. The stress fiber formation caused by Angptl3 may play a role in protecting podocytes from damage, but this effect is weak and short-lived. The following long-term lamellipodia formation caused by Rac1 stimulation can increase the motility of podocytes, which may aggravate foot process effacement and result in proteinuria.

Since integrin *α*_*V*_ *β*_3_ is the main receptor for Angptl3-mediated F-actin rearrangement, and Angptl3 binds to integrin *α*_*V*_ *β*_3_ through its FLD, this Angptl3 domain is the key component that causes Rac1 and RhoA activation and podocyte F-actin rearrangement. Our result may provide a new method of protecting podocytes and preventing F-actin rearrangement and foot process effacement in proteinuric kidney diseases.
